# Eco-Friendly Engineered Nanomaterials Coupled with Filtering Fine-Mesh Net as a Promising Tool to Remediate Contaminated Freshwater Sludges: An Ecotoxicity Investigation

**DOI:** 10.3390/nano13030396

**Published:** 2023-01-18

**Authors:** Patrizia Guidi, Margherita Bernardeschi, Mara Palumbo, Isabella Buttino, Valentina Vitiello, Vittoria Scarcelli, Gianluca Chiaretti, Andrea Fiorati, David Pellegrini, Lorenzo Pontorno, Lisa Bonciani, Carlo Punta, Ilaria Corsi, Giada Frenzilli

**Affiliations:** 1 Department of Clinical and Experimental Medicine, Section of Applied Biology and Genetics, and INSTM Local Unit, University of Pisa, 56126 Pisa, Italy; 2 Italian Institute for Environmental Protection and Research (ISPRA), Via del Cedro, 38, 57123 Livorno, Italy; 3 Department of Chemistry, Materials, and Chemical Engineering “G. Natta”, and INSTM Local Unit, Politecnico di Milano, 20131 Milano, Italy; 4 Biochemie Lab. S.r.l, Via di Limite 27G, 50013 Campi Bisenzio, Italy; 5 Department of Physical, Earth and Environmental Sciences, and INSTM Local Unit, University of Siena, 53100 Siena, Italy

**Keywords:** eco-friendly nanomaterials, nanoremediation, cellular responses, DNA damage, chromosomal alterations, acute toxicity

## Abstract

The use of eco-friendly engineered nanomaterials represents a recent solution for an effective and safe treatment of contaminated dredging sludge. In this study, an eco-designed engineered material based on cross-linked nanocellulose (CNS) was applied for the first time to decontaminate a real matrix from heavy metals (namely Zn, Ni, Cu, and Fe) and other undesired elements (mainly Ba and As) in a lab-scale study, with the aim to design a safe solution for the remediation of contaminated matrices. Contaminated freshwater sludge was treated with CNS coupled with a filtering fine-mesh net, and the obtained waters were tested for acute and sublethal toxicity. In order to check the safety of the proposed treatment system, toxicity tests were conducted by exposing the bacterium *Aliivibrio fischeri* and the crustacean *Heterocypris incongruens*, while subtoxicity biomarkers such as lysosomal membrane stability, genetic, and chromosomal damage assessment were performed on the freshwater bivalve *Dreissena polymorpha*. Dredging sludge was found to be genotoxic, and such genotoxicity was mitigated by the combined use of CNS and a filtering fine-mesh net. Chemical analyses confirmed the results by highlighting the abetment of target contaminants, indicating the present model as a promising tool in freshwater sludge nanoremediation.

## 1. Introduction

Contamination of water bodies is known to compromise ecosystem health, and urgent actions need to be taken to develop sustainable and eco-friendly solutions toward zero pollution [1,2,3]. Nanoremediation is a quite recent remediation technique relying on the use of engineered nanomaterials (ENMs) to clean up polluted matrices [4,5]. It has attracted more and more attention in the last decade, but it still needs to be elaborated, with a special focus on safety, due to the uncertainty related to ENMs’ fate and (eco)toxicity [6,7,8,9]. In fact, if the success of nanoremediation is mainly related to the peculiar properties of ENMs, including their high surface area and high reactivity, making them able to sequester, degrade, or transform pollutants, the same properties could represent an issue by considering the risks associated to their interaction with surrounding ecosystems [10,11]. As a consequence, while many ENMs have been proposed for the remediation of contaminated water and soils, their safety is still under debate. For example, zero-valent iron nanoparticles (nZVI), recently proposed for nanoremediation oxidative processes, have shown to be (eco)toxic [12], while nano-zinc, carbon nanotubes, and nano-titanium oxides, despite their great potential for efficient decontamination, are known to pose serious risks to aquatic life [10]. In order to overcome these issues and concerns, the eco-design of ENMs, which includes a green and sustainable synthesis combined with advanced ecotoxicological tools for risk assessment, is seen as a promising solution [13,14,15,16,17]. 

In this context, in recent years, we have reported the eco-design and synthesis of a new class of cross-linked nanocellulose (CNS), characterized by nanoporosity [18,19], which was safe and highly effective for water decontamination from heavy metals and organic molecules [20,21,22]. CNS were already proven to be safe for aquatic species by ecotoxicity assessment either with marine or freshwater species [10,23,24]. Specifically, in the framework of the Nanomaterials for Remediation of Environmental Matrices associated to Dewatering (NANOBOND) POR CReO FESR project, we have conceptualized the combination of CNS with a filtering fine-mesh net for the treatment of contaminated dredged sludge, in order to achieve the direct decontamination of outflowing water [16]. 

Herein, we report, for the first time, the results obtained at the laboratory scale by testing this approach on a real environmental matrix, characterized by the presence of different inorganic pollutants (mainly Zn, Ni, Cu, Fe, Ba, and As), with the final aim of both validating CNS decontamination efficiency in a simulated scenario and transferring the information obtained in previous single-contaminant exposure studies [10,24] to a laboratory scale prototype for freshwater sludge nanoremediation. Moreover, the coupling of both technologies (CNS and fine-mesh net) avoids the direct release of CNS into an aqueous matrix upon application for sludge treatment. 

To corroborate the effectiveness of the suggested remediation strategy, chemical analyses on both polluted freshwater sludge alone and treated with CNS, and on water from sludge dewatering, were set up. Furthermore, to ascertain that the proposed remediation trial was effective, and the resulting waters were not toxic, a battery of ecotoxicity tests and sublethal biomarker responses on model freshwater species were applied. Acute toxicity tests were conducted by exposing the bacterium *Aliivibrio fischeri* and the crustacean *Heterocypris incongruens* to freshwater extracted from wet contaminated sludge by filtration with and without CNS addition and sediment trapped in the net, respectively. Moreover, genotoxicity and cytotoxicity, induced by water obtained from filtering, were assessed on the hemocytes of exposed specimens of *Dreissena polymorpha*, the most common bivalve for genotoxicity assessments in freshwater environments [25]. Genotoxic effects were evaluated by the comet assay to detect DNA primary damage and by the cytome assay to assess chromosomal damage, widely applied to freshwater organisms [26,27]. The neutral red retention time (NRRT) assay was used to assess lysosomal membrane stability. 

## 2. Materials and Methods

### 2.1. Chemicals and Devices

All the reagents for CNS synthesis and artificial freshwater (AFW) constituents were purchased from Sigma-Aldrich (Milano, Italy), except for cotton linters, which were provided by Bartoli Spa (Capannori, Lucca, Italy). All the reagents for the comet assay were purchased from Sigma-Aldrich (Steinheim, Germany). Phosphate-buffered solution (PBS) and lead citrate constituents were purchased from Carlo Erba Reagenti (Milan, Italy). Ethanol and HCl were purchased from PanReac AppliChem (Barcelona, Spain). Giemsa was purchased from Titolchimica S.p.A (Rovigo, Italy). Inductively coupled plasma–optical emission spectrometry (ICP-OES) analysis was conducted by using a Perkin Elmer Optima 8300 (PerkinElmer, Inc., Waltham, MA, USA) equipped with a CrossFlow nebulizer and a Scott spray chamber, followed by a standard quartz torch. The instrument calibration was performed by dilution of analytical standard (FLUKA) with MilliQ^®^ water (Merck Life Science, Milano, Italy); to each analyzed sample, Y (2 mg/L) was added as an internal standard. The freeze-dried *A. fischeri* bacteria and *Heterocypris incongruens*, “Ostracodtoxkit F”, were purchased by Ecotox LDS S.r.l. (Milan, Italy). Metals analysis evaluation was set up by ICP-MS 7900 (Agilent, Santa Clara, CA, USA).

### 2.2. Preparation and Characterization of CNS

CNS were produced as reported in a previous paper [21]. In a first step, cellulose was oxidized and mechanically treated to produce TOCNF (oxidation degree of ~1.5 mmolCOOH/gTOCNF) [28]. Briefly, cellulose (190 g), TEMPO (2.15 g, 13.8 mmol), KBr (15.42 g, 129 mmol), and NaClOaq (12.5% *w*/*w*, 437 mL) were added to 5.7 L of deionized water under vigorous mechanical stirring. pH was maintained in the range of 10.5–11 by dripping 4 N NaOHaq. After 16 h, TOCNF were aggregated by using 37% HClaq, filtered and washed with deionized water up to reach a neutral pH, and further suspended in deionized water (3% *w*/*v*) in the presence of stoichiometric amounts of NaOH. The dispersion was ultrasonicated at 0 °C for 30 min. After acidification (12 M HClaq), TOCNF were filtered, washed with deionized water (450 mL × 3) up to neutrality, and were then redispersed in 500 mL of an aqueous solution containing 25 kDa bPEI (84 g) and citric acid (CA) (23.8 g) [29]. The resulting homogeneous hydrogel was transferred to a 24 multiwell plate, frozen at −35 °C, freeze-dried for 48 h, and heated in an oven at 102 °C for 16 h [29]. Finally, CNS were ground in a mortar and then washed with water (6 × 150 mL).

### 2.3. Exposure Water Set-Up

**Figure 1:**
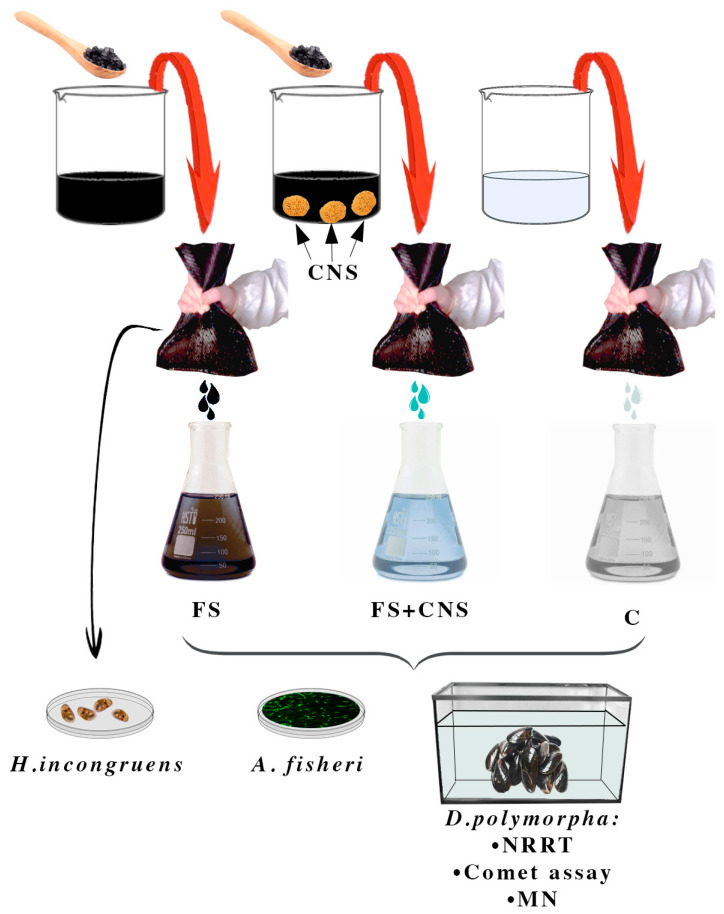
Experimental design. Set-up of three treatment groups: AFW as control (C); water from the industrial canal sludge filtration (FS); water obtained by filtration of sludge previously enriched in CNS (FS + CNS). Filtration was achieved by passing twice the sludge through a fine-mesh net.

In order to obtain 2 L of mixture 15% in weight, freshwater sludge taken from the Caligi industrial canal (Tuscany, Italy) was added to artificial freshwater (AFW), obtaining 2 replicates, 2 L each. At the end of the procedure, one sample was enriched in CNS (1.25 gL^−1^). Both samples were stirred for 2 h, after which anionic and poliaminic flocculants at the final concentration of 5‰ were added. Finally, samples were left to decant overnight. The two mixtures of sludge and water and an artificial freshwater control sample were filtered twice through the textile providing three water samples, which were used to set up both acute ecotoxicity and sublethal tests. In summary, the experimental design involved three treatment groups: AFW as control (C), water from the industrial canal sludge filtration (FS), and water obtained by filtration of sludge previously enriched in CNS (FS + CNS) (Figure 1). 

### 2.4. Freshwater Sludge Chemical Analyses

Chemical analyses were performed on water samples resulting from fine-mesh net filtration collected immediately before the bivalve exposure. Three water samples (C, FS, FS + CNS) and two sediment samples trapped in the fine mesh (S, S + CNS) were analyzed for metal concentrations through inductively coupled plasma–optical emission spectrometry (ICP-OES).

### 2.5. Ecotoxicity Assessment

#### 2.5.1. Acute Toxicity Test with *Aliivibrio fischeri*

The effect of the decontamination of industrial canal sludge by CNS in combination with a fine-mesh net has been verified by an acute toxicity test on *Aliivibrio fischeri* performed as reported by Guidi et al. [24] following the standard protocol UNI EN ISO (ISO 11348-3:2019) [30]. This test is based on the luminescence naturally emitted by the marine bacterium *A. fischeri* after its exposure to a toxic substance in the water matrix. Briefly, bacteria were added to each sample and incubated at 15 °C using a Microtox M500 luminometer. Seven dilutions and three replicates were performed for each sample. Bacteria light emission was recorded at the beginning of incubation and after 5, 15, and 30 min of exposure. Bacteria added to a toxic-free solution represented the control group. Data were analyzed using MicrotoxOmni™ software (version 4.2, Modern Water, London, UK), which calculates EC20 and EC50, concentrations corresponding to 20% and 50% reduction of bioluminescence compared to the control, respectively. Toxic effects were considered when the EC20 was <90% [30,31,32]. The maximum effect percentage recorded at the highest concentration of samples, calculated as mean ± SD of the exposure time 5, 15, and 30 min, was detected by the software when the EC20 value was ≥90%.

#### 2.5.2. Acute Toxicity Test with *Heterocypris incongruens*

The benthic ostracod crustacean *Heterocypris incongruens* was directly exposed to sediment (S, S + CNS) according to the ISO 14371:2012 protocol [33]. Mortality was expressed as mean percentage with Abbott’s correction [34], which normalizes the effects compared to a standard sediment. Toxicity was considered when the mortality of the exposed organisms was >20% [31,32,33,34,35], and mortality of the controls exposed to a nonpolluted standard sediment was <20%.

#### 2.5.3. Sublethal Toxicity with the Freshwater Bivalves *D. polymorpha*: Sampling, Maintenance Condition, and Laboratory Exposure

Adult specimens of zebra mussel (*Dreissena polymorpha*) (average valve length 2 ± 0.5 cm) were collected from Bilancino Lake, a pristine area in Tuscany (Florence, Italy), and transported to the laboratory in original fresh lake water. Mussels were placed 2 days before experiments in a 10 L aerated tank with artificial freshwater (AFW) obtained by mixing distilled (50%) and dechlorinated tap water (50%) for the acclimatization period. Water temperature was 18 ± 1 °C, and a natural photoperiod was maintained; pH values were 7.70 ± 0.30.

After the acclimatization time, mussels were placed in glass aerated tanks, each of which was equipped with an oblique glass sheet. Specimens of zebra mussels were distributed in three different glass tanks each representative of one of the three experimental groups: control (C), water from the industrial canal sludge filtration (FS), and water obtained by filtration of sludge previously enriched in CNS (FS + CNS). 

For each aquaria treatment, at least 25 zebra mussels were exposed for 48 h. Zebra mussels were not fed during the experiments, and only specimens that were able to reattach themselves by their byssus filaments on glass sheets immersed in water were used for the research, as suggested by Binelli and collaborators [36]. At the end of the exposure time, approximately 150 µL of zebra mussel hemolymph was gently aspirated with a syringe (25G5/8) containing 100 µL of PBS from the posterior adductor muscle sinus. Individual hemolymph samples were poured from the syringe without a needle on a single tube, and then hemocyte suspensions were processed for NNRT, comet, and cytome assays [37].

#### 2.5.4. Sublethal Toxicity with the Freshwater Bivalves *D. polymorpha*: Viability Assessment

The neutral red retention time (NRRT) assay is a cheap and rapid measurement of lysosomal membrane destabilization [38] and was performed on *D. polymorpha* hemocytes according to Guidi and collaborators [39] as an index of cell viability. 

#### 2.5.5. Sublethal Toxicity with the Freshwater Bivalves *D. polymorpha*: Comet Assay

The comet assay was carried out on hemocytes from 10 specimens per tank at the end of the exposure, according to Singh et al. [40] and Møller et al. [41], with slight modifications [42], and it was performed only with cell populations that showed a viability >90% to avoid false-positive results. During the hemolymph collection, individual cell suspensions were stored at +4 °C in the dark, and then samples were immediately centrifuged for 10 min at 125× *g*. The cell pellet was embedded in 75 µL of freshly made 0.5% LMA and spread on microscopy glass slides, precoated with a layer of 1% NMA. The second layer of agarose polymerization was allowed for 5 min on metal trays at +4 °C, and then an additional layer of 85 µL of 0.5% LMA was added. Following agarose solidification at room temperature, slides were immersed in freshly made lysing solution (10 mM Tris, 2.5 M NaCl, 1% Triton X × 100, 0.1 M EDTA, and 10% DMSO, pH 10) for at least 1 h. To allow DNA unwinding in alkaline conditions, a horizontal gel electrophoresis chamber was used to incubate slides for 10 min with fresh electrophoresis buffer (0.075 M NaOH, 1 mM EDTA, pH ≥ 13). Electrophoresis was performed at 25 V, 300 mA, for 5 min. To neutralize the pH and allow DNA staining, at the end of the electrophoresis run, slides were washed three times (5 min each) with a neutralization solution (Tris-HCl, pH 7.5). Slides were stained with ethidium bromide and scored under a fluorescence microscope (400×). An image analyzer (Kinetic Imaging, Ltd., Liverpool, UK, Komet, Version 5) was used, and the parameter chosen to quantify the amount of DNA damage was the percentage of DNA migrated into the comet tail (% tail DNA) [43]. All steps were conducted in the dark. At least 50 randomly chosen nuclei per slide and 2 slides per specimen, with 10 bivalves per treatment tank, were scored for a total of 100 nuclei per organism, and the mean was calculated.

#### 2.5.6. Sublethal Toxicity with the Freshwater Bivalves *D. polymorpha*: Cytome Assay 

The genotoxic effects were evaluated at the chromosomal level by the micronuclei and nuclear abnormalities frequency assessment. The cytome assay was carried out on hemocytes according to Scarpato et al. [44], with slight modifications [24]. Cells were prefixed for 20 min at room temperature in a 5% acetic acid, 3% methanol, and 92% PBS 20‰ solution and centrifuged for 10 min at 125× *g*. The supernatant was removed, and 1 mL of fixative solution (1:7 acetic acid and ethanol) was added twice. After the last fixation, cells were centrifuged (10 min at 125× *g*), spread onto slides (two slides per mussel), air dried, and stained with 3% Giemsa solution for 10 min. Cells with a well-preserved cytoplasm were scored (500 per slide) under a light microscope to determine the frequency of micronuclei and nuclear abnormalities according to the criteria proposed by Fenech [45]. The presence of hemocytes with a morphologically altered nucleus was scored and reported as the frequency of total nucleus abnormalities (NA). The NA set includes nuclear blebs, nuclear buds, notched nucleus, lobed nucleus, and cells with nuclear bridges [46]. A total of 10 specimens, 2 slides per specimen, and 500 cells per slide were scored for each experimental group.

### 2.6. Statistical Analysis

Results obtained are represented as mean ± SD from at least 10 specimens. Data were analyzed by the multifactor analysis of variance (MANOVA). The multiple range test (MRT) was performed in order to detect differences among experimental groups. For all data analyses, statistical significance was set at *p* < 0.05.

## 3. Results

### 3.1. Water Exposure and Sediment Characterization

Metal concentrations in the three experimental water exposure groups and in the sediments trapped in the net are reported in Table 1.

### 3.2. Acute Toxicity Test with Aliivibrio fischeri

In Table 2, the results of the ecotoxicology test with Microtox^®^ carried out with the aqueous fraction obtained from sludge mechanically filtered through the fine-mesh net (FS) and with water obtained by sludge enriched in CNS filtered through the filtering fine-mesh net (FS + CNS) are reported.

Both FS and FS + CNS samples did not induce any toxic effect on bacteria; EC20 values, calculated for each incubation time, were over 90%; the max effect percentage, index of bioluminescence decrease, calculated as the mean between 5, 15, and 30 min data, was below 20%. The addition of the industrial canal sludge with the anionic flocculants at 5‰ (FS sample) did not induce any significant effect on the bioluminescence of bacteria exposed to the water leaving the filtering fine-mesh net. Similarly, the water passing through the filtering fine-mesh net, obtained from the industrial canal sludge added with anionic flocculants and cellulose-based nanostructured materials (FS + CNS sample), did not significantly modify *A. fischeri* bioluminescence, even at the longest exposure time.

### 3.3. Sludge Toxicity with Heterocypris incongruens

Table 3 shows the results of the ecotoxicology test with the crustacean *Heterocypris incongruens* carried out with standard sediment (Reference Control) and the industrial canal sediment, with and without CNS (S + CNS and S).

At the end of the exposure, a low mortality percentage (3.33) was recorded for those organisms incubated in a standard sediment (control group), determining the validation of this test. The exposure of organisms to sediment (S) induced a mortality of 13.33%, which corresponds to 11.54% after Abbott’s correction, whereas a certain degree of mortality (25%) was recorded in organisms incubated for 6 days in sediment enriched in CNS (S + CNS). Abbott’s correction brings the mortality recorded with this experimental group to 28.89%.

### 3.4. Viability Assessment on D. polymorpha

**Figure 2:**
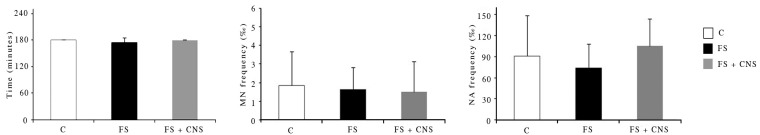
Results from cellular biomarker analysis in zebra mussel hemocytes after the exposure to different experimental groups. Neutral red retention time results are expressed in minutes (min); micronucleated cells (MN), and total nuclear abnormalities (NA) are expressed as frequency (‰). Control group (artificial freshwater AFW), water obtained by sludge filtration (FS) and water obtained by sludge enriched in CNS filtered by fine-mesh net (FS + CNS). Data are expressed as mean ± SD.

Concerning lysosomal membrane stability, no statistically significant differences were observed among specimens exposed to the treatment waters both with and without CNS (FS; FS + CNS) in comparison with the control group, as reported in Figure 2.

### 3.5. Comet Assay on D. polymorpha

**Figure 3:**
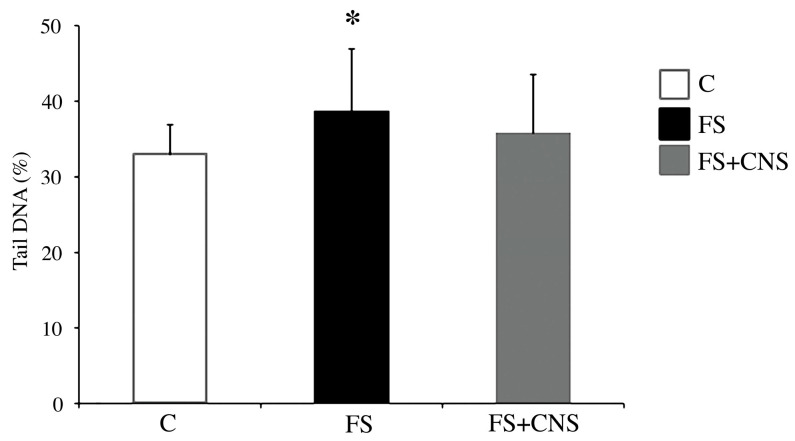
DNA primary damage (% tail DNA) in zebra mussel hemocytes after 48 h exposure to the following experimental groups: C (control); FS (water obtained after sludge filtration); water obtained by sludge enriched in CNS filtered by fine-mesh net (FS+CNS). Results are shown as mean ± SD. (*) indicates significant differences with respect to the control group (C) (*p* < 0.05).

The comet assay results (Figure 3) showed a statistically significant increase (*p* < 0.05) in DNA damage in specimens of *D. polymorpha* exposed to water obtained after sludge filtration (FS group) in comparison with specimens belonging to the control group, as shown in Figure 3. The FS + CNS exposure group did not show any statistically significant difference from the control group in terms of DNA primary damage.

### 3.6. Cytome Assay on D. polymorpha

No statistically significant differences were observed in terms of micronucleated cells (MN) and total nuclear abnormalities (NA) among specimens exposed to the water obtained by sludge filtered by the fine-mesh net both with and without CNS in comparison with the control group. Data are reported in Figure 2.

## 4. Discussion

The present work aimed at evaluating the efficiency of reducing metal contamination in water obtained by the filtration of sludge treated with CNS. It turned out that for most of the contaminants, it was possible to move from high concentrations up to levels acceptable for drinking water (e.g., in the case of arsenic, iron, vanadium, and zinc). 

After the positive feedback on the metal adsorbent effectiveness of the technology here proposed, it was considered to be appropriate in evaluating the biological effects through an ecotoxicological approach. In this way, this innovation becomes more useful than destructive, as suggested in the concluding remarks of Hussain and co-workers [47]. For this reason, the decreasing adverse effects of contaminated sludge and wastewaters coming from polluted sediment dewatering were assessed. The combined technology has been proposed as a new eco-friendly tool for the effective application of nanotechnology on sustainable environmental remediation following some of the proposals reported by Ganie et al. [15], to sustain the resilience of our planet. Conventional in situ treatment systems, such as thermal treatment, air sparging, chemical oxidation, and bioremediation, often coupled with on-site pump-and-treat processes [48], present evident limits: expensiveness, only partial effectiveness, and time-consuming [19]. For these reasons, some biological implications and welfare hazards may limit the wide use of nanomaterials for ecological remediation. 

The proposed contaminated sludge treatment system, if translated from the laboratory scale to restoration activity, might represent a new in situ clean-up method. The coupled approach could be cheaper, safer, and more efficient compared to conventional techniques, allowing the processing of contaminated matrices without moving them to treatment plants, thus reducing logistic and time costs [47].

### 4.1. Efficacy in Terms of Adsorbent Capacity of CNS

A key point of the present remediation technology is based on the adsorbent capacity of the bio-based and nontoxic nanomaterial. Since biocompatibility and biodegradability are two essential components for the use of polymer nanocomposites, as discussed by Sun et al. [49], it is worth noting how the CNS here applied were obtained by cellulose as a sustainable and renewable raw starting material. CNS were produced through sustainable synthetic approaches [21], and they do not exert any toxicity even when combined with filtering fine-mesh net pressing, as supported by the present results. In previous freshwater in vivo studies [10,24], cellulose-based nanosponges were found to be safe for the biota and effective in facing pollutant insult of zinc- and cadmium-contaminated artificial water, proving their absorbent ability. Similarly, data reported in Table 1 concerning water extracted through fine-mesh filtering from contaminated wet sludge treated with CNS confirm the capacity of CNS to sequester metals from contaminated freshwater sludge. These results suggest that the proposed combined techniques could be useful in the remediation of metal-contaminated freshwater sludges.

### 4.2. Safety in Terms of Acute Toxicity (Aliivibrio fischeri and Heterocypris incongruens)

The potential biological effect of the proposed remediation technique has been verified by the use of acute toxicity tests on *Aliivibrio fischeri* and *Heterocypris incongruens.* Results concerning the *A. fischeri* experiment supported the suitability and safety of the proposed technique. On the other hand, the mortality of *H. incongruens* incubated in the sediment enriched in CNS and squeezed by the fine-mesh net (S + CNS) exceeded, albeit slightly, the threshold value of 20%, indicating this sample as toxic. In fact, the cut-off value of 20% is considered the limit level to discriminate between nontoxic and toxic samples. 

Comparing the chemical data related to freshwater sediments treated and not treated with CNS after dewatering, no substantial differences between the two experimental groups (S, S + CNS) were observed in terms of heavy metal concentration. CNS, able to adsorb heavy metals present in the wet sludge not removed from the dehydrated matrix, possibly caused the mild toxicity exerted by the solid fraction enriched in CNS retained by the fine-mesh net on *H. incongruens*. This data underlines the importance of the association of the two technologies in nanoremediation activities to prevent the dispersion in the environment of the nanomaterial following its contaminant absorbent action. 

### 4.3. Safety in Terms of Acute Sublethal Toxicity with the Freshwater Bivalves D. polymorpha

In the present experimental set-up, the exposure to waters obtained from the filtering fine-mesh net sludge enriched in CNS did not induce any loss of lysosomal membrane stability, chromosomal damage, and nuclear morphological alterations in *D. polymorpha* with respect to the control group. DNA primary damage levels detected in the FS experimental group were reduced in specimens exposed to the waters obtained by the association between fine-mesh net filtering and CNS absorbing action. The lower DNA insult detected in the animals exposed to waters obtained by combining the sludge filtration and the CNS sludge treatment (FS + CNS) is probably due to the pollutant sequestering activity of CNS. The presence of a DNA primary damage assessed in the FS experimental group and not paralleled by chromosomal damage can be ascribable to the fact that the comet assay and micronucleus test are complementary tests in assessing genotoxicity, as they can detect different aspects of genotoxicity of pollutants [25,50,51]. It has been reported that different model organisms exposed to heavy metals showed increased levels of DNA primary damage evaluated by the comet assay even in the absence of chromosomal damage [52,53,54].

Genotoxicity results support the suitability and safety of CNS coupled with a filtered mesh net to mitigate the effect exerted by freshwater obtained by contaminated sludge filtration.

## 5. Conclusions

These results are encouraging to contribute to developing sustainable strategies to face water quality and water scarcity problems. The present treatment system could be proposed as a new in situ clean-up method if translated from the laboratory scale to decontamination activity, taking into account all the aspects related to the complexity of remediation in the real environmental field. In fact, for a complete sediment treatment, the contaminant-soaked nanomaterials should be removed from the sediment before speaking in terms of decontaminated sludge. However, in our experimental model, the contaminated sludge with added CNS remains confined within the net and is not released into the environment. 

The results presented here show how the water quality obtained from dehydrated sludge has been improved by treating contaminated freshwater sludge through resuspension with nanomaterials and fine-mesh filtration. In conclusion, the present data support the combination of draining net technology with the addition of cellulose-based nanostructured materials that appears as an interesting tool to obtain water, which can be potentially released into the environment. 

## Figures and Tables

**Table 1:** Concentration of metals in exposure waters and sediments. Water samples: control group (C), outflowing water obtained by sludge filtration (FS), and outflowing water obtained by sludge enriched in CNS filtered by fine-mesh net (FS + CNS). Sediment samples: sediment trapped in the net (S) and sediment enriched in CNS trapped in the net (S + CNS).

Matrices		Water (mg/L)		Freshwater Sediment (mg/L)	
Substance	C	FS	FS + CNS	S	S + CNS
TOC	5.16 mg/L	86.6	85.0	1.2%	1.1%
**Contaminant**		**(µg/L)**		**(mg/kg)**	
Aluminum	17.2	<1.0	<1.0	18,000	16,000
Arsenic	<1.0	22.6	3.96	3.85	3.13
Barium	26.2	163	56.7	115	112
Cadmium	<0.5	<0.5	<0.5	15.9	15.8
Chrome	<1.0	2.13	<1.0	70.2	68.5
Iron	15.5	1390	139	23,486	21,982
Mercury	<0.1	<0.1	<0.1	0.34	0.32
Nickel	<1.0	19.6	1.5	47.1	36.8
Lead	<1.0	1.0	<1.0	47.2	41.6
Copper	9.0	2.1	<1.0	532	500
Vanadium	<1.0	7.6	<1.0	31.6	30.5
Zinc	15.1	14.2	<1.0	179	175

**Table 2:** EC20 values after 5, 15, and 30 min of incubation and maximum effect percentage (mean ± SD) at the highest concentration of samples.

	EC20 (%)			Max Effect (%)
	5 min	15 min	30 min	Mean ± SD
Control	>90	>90	>90	7.18 ± 1.01
FS	>90	>90	>90	6.62 ± 7.29
FS + CNS	>90	>90	>90	−5.37 ± 11.99

**Table 3:** *Heterocypris incongruens* bioassay test. Percentage of mortality and Abbott’s corrected mortality recorded.

	Mortality (%)	Abbott’s Corrected Mortality (%)
Reference Control	3.33	-
S	13.33	11.54
S + CNS	25.00	28.89

## Data Availability

Not applicable.
